# A Noncoding Point Mutation of *Zeb1* Causes Multiple Developmental Malformations and Obesity in Twirler Mice

**DOI:** 10.1371/journal.pgen.1002307

**Published:** 2011-09-29

**Authors:** Kiyoto Kurima, Ronna Hertzano, Oksana Gavrilova, Kelly Monahan, Karl B. Shpargel, Garani Nadaraja, Yoshiyuki Kawashima, Kyu Yup Lee, Taku Ito, Yujiro Higashi, David J. Eisenman, Scott E. Strome, Andrew J. Griffith

**Affiliations:** 1 Otolaryngology Branch, National Institute on Deafness and Other Communication Disorders, National Institutes of Health, Rockville, Maryland, United States of America; 2 Department of Otorhinolaryngology–Head and Neck Surgery, University of Maryland, Baltimore, Maryland, United States of America; 3 Mouse Metabolism Core Laboratory, National Institute of Diabetes and Digestive and Kidney Diseases, National Institutes of Health, Bethesda, Maryland, United States of America; 4 Department of Perinatology, Institute for Developmental Research, Aichi Human Service Center, Kasugai, Japan; The Jackson Laboratory, United States of America

## Abstract

Heterozygous Twirler (*Tw*) mice develop obesity and circling behavior associated with malformations of the inner ear, whereas homozygous *Tw* mice have cleft palate and die shortly after birth. Zeb1 is a zinc finger protein that contributes to mesenchymal cell fate by repression of genes whose expression defines epithelial cell identity. This developmental pathway is disrupted in inner ears of *Tw/Tw* mice. The purpose of our study was to comprehensively characterize the Twirler phenotype and to identify the causative mutation. The *Tw/+* inner ear phenotype includes irregularities of the semicircular canals, abnormal utricular otoconia, a shortened cochlear duct, and hearing loss, whereas *Tw/Tw* ears are severely malformed with barely recognizable anatomy. *Tw/+* mice have obesity associated with insulin-resistance and have lymphoid organ hypoplasia. We identified a noncoding nucleotide substitution, c.58+181G>A, in the first intron of the *Tw* allele of *Zeb1* (*Zeb1^Tw^*). A knockin mouse model of c.58+181G>A recapitulated the *Tw* phenotype, whereas a wild-type knockin control did not, confirming the mutation as pathogenic. c.58+181G>A does not affect splicing but disrupts a predicted site for Myb protein binding, which we confirmed *in vitro*. In comparison, homozygosity for a targeted deletion of exon 1 of mouse *Zeb1*, *Zeb1^ΔEx1^*, is associated with a subtle abnormality of the lateral semicircular canal that is different than those in *Tw* mice. Expression analyses of E13.5 Twirler and *Zeb1^ΔEx1^* ears confirm that *Zeb1^ΔEx1^* is a null allele, whereas *Zeb1^Tw^* RNA is expressed at increased levels in comparison to wild-type *Zeb1*. We conclude that a noncoding point mutation of *Zeb1* acts via a gain-of-function to disrupt regulation of *Zeb1^Tw^* expression, epithelial-mesenchymal cell fate or interactions, and structural development of the inner ear in Twirler mice. This is a novel mechanism underlying disorders of hearing or balance.

## Author Summary

Twirler (*Tw*) mice have a combination of abnormalities that includes cleft palate, malformations of the inner ear, hearing loss, vestibular dysfunction, obesity, and lymphoid hypoplasia. In this study, we show that the underlying mutation affects the *Zeb1* gene. *Zeb1* was already known to encode a protein normally expressed in mesenchymal cells, where it represses expression of genes that are uniquely expressed in epithelial cells. The *Tw* mutation is a rare example of a single-nucleotide substitution in a region of a gene that does not encode protein, promoter, or splice sites, so we engineered a mouse model with the mutation that confirmed its causative role. The *Tw* mutation disrupts a consensus DNA binding site sequence for the Myb family of regulatory proteins. We conclude that this mutation leads to abnormal expression of *Zeb1*, structural malformations of the inner ear, and a loss of hearing and balance function. A similar mechanism may underlie other features of Twirler, such as obesity and cleft palate.

## Introduction

Twirler (*Tw*) spontaneously arose in a crossbred stock of mice segregating multiple recessive mutant alleles [1]. Heterozygous *Tw* mice develop obesity after three months of age, and exhibit stereotypic behavior that includes waltzing, spinning, and horizontal head-shaking [1]. This behavior is thought to result from malformed vestibular labyrinths that include hypomorphic or absent lateral semicircular canals, irregular contours of the anterior and posterior semicircular canals, and absent otoconia in the utricle and saccule [1]. In contrast, all homozygous *Tw* mice are born with cleft palate and die soon after birth [1].

*Tw* is located on proximal chromosome 18 but the causative mutation has not been identified [1], [2]. A transgene insertional mutant, Tg9257, exhibits a similar inner ear phenotype and is also located on proximal chromosome 18, raising the possibility that these phenotypes are allelic [3]. However, complementation testing is inconclusive [3]. Similarly, the *Irxl1* gene, located within a broad critical map interval for *Tw* and expressed in developing palate, has also been ruled out as a candidate for *Tw* [4].

*Zeb1* is also located on proximal chromosome 18 and encodes a transcription factor, Zeb1, that binds E-box-like elements to either repress [5], [6], or activate transcription [7]–[9]. Mice that are homozygous for a targeted deletion of exon 1 of *Zeb1* (*Zeb1^ΔEx1^*) die soon after birth with cleft palate, limb defects and other skeletal abnormalities, and T-cell deficiency [10], whereas heterozygous *Zeb1^ΔEx1/+^* mice are viable and adult females show increased adiposity [11]. This partial phenotypic overlap with Twirler does not include stereotypic vestibular behavior or inner ear malformations, although these were likely not examined in *Zeb1^ΔEx1^* mice. Ectopic expression of Zeb1 in neoplastic epithelium has been implicated in the epithelial-to-mesenchymal transition (EMT) leading to local tumor invasion and metastasis [12]. In normally developing mesenchymal tissue, Zeb1 is thought to repress epithelial-specific genes such as E-cadherin and activate mesenchyme-specific genes such as collagen, smooth muscle actin and myosin [9]. Genome-wide expression profiling reveals a probable similar role for Zeb1 in the regulation of gene expression in developing mouse inner ear mesenchyme [13]. In humans, heterozygous mutations of *ZEB1* cause posterior polymorphous corneal dystrophy, characterized by an epithelial transition and abnormal proliferation of corneal endothelium [14]. *Zeb1^ΔEx1/+^* mice also show corneal abnormalities and further implicate Zeb1 in the suppression of an epithelial phenotype [15].

In the current study we show that Twirler is caused by a noncoding point substitution in the first intron of *Zeb1*. The mutation does not affect splicing, but does disrupt a consensus binding site sequence for Myb proteins [16]. The maintenance of inner ear mesenchyme- and epithelium-specific gene expression is disrupted in Twirler inner ears [13], demonstrating a novel mutation and developmental mechanism for the pathogenesis of hearing or balance disorders.

## Results

### Lymphoid phenotype of Twirler mice

Heterozygous *Tw*/+ adult mice had smaller spleens (38±2 mg vs. 68±7 mg, P<0.013) in comparison to wild type littermates. *Tw/+* thymi were also smaller although the difference was not significant (13±2 mg vs. 31±6 mg, P<0.06). *Tw/+* mice had lower counts of white blood cells (1×10^3^/µl vs. 7.2×10^3^/µl, P<0.0001), lymphocytes (0.5×10^3^/µl vs. 5.9×10^3^/µl, P<0.0004) and polymorphonuclear neutrophils (0.4×10^3^/µl vs. 1.3×10^3^/µl, P<0.04). No abnormalities were found in other adult *Tw/+* tissues. Histopathological examination of P0 animals revealed no abnormalities in the thymus or spleen of wild type, *Tw*/+ or homozygous *Tw/Tw* mice. *Tw/Tw* mice had cleft palates.

### Obesity and metabolic phenotype of Twirler mice

**Figure 1:**
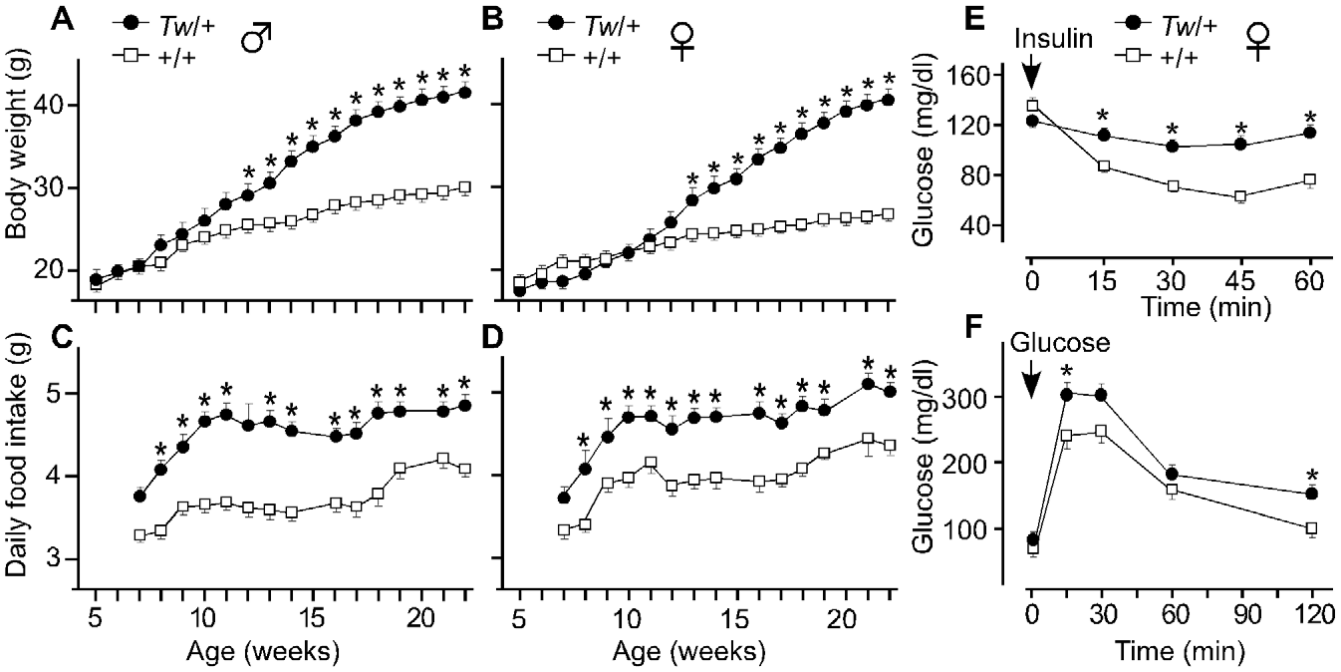
Body weight, daily food intake, and insulin and glucose tolerance of Twirler mice. Body weight was measured weekly in males (A) and females (B). (n = 6 for each sex and each genotype). Food intake was measured weekly to calculate average daily intake for males (C) and females (D). *Tw/+* males and females show increased body weight after 12 and 13 weeks of age, respectively. Both *Tw*/+ males and females show increased food intake at most tested ages. Serum glucose levels are shown for *Tw*/+ (n = 8) and wild type (n = 6) female mice injected with insulin (E) or glucose (F) at 15 weeks of age. Mean body weights were 28.5 g and 22.4 g for *Tw/+* and wild type mice, respectively. *Tw/+* mice show insulin resistance (E). In the glucose tolerance test, the area under the curve (AUC) is significantly higher in *Tw/+* mice compared with wild type mice (p = 0.039). The vertical bars indicate the standard error of the mean (SEM). Asterisks indicate P<0.05 in an unpaired Student's *t* test.

There was no significant difference in average body weight between *Tw/+* and wild type littermates of either sex until 12 weeks of age (Figure 1A and 1B). Beginning at seven weeks of age, *Tw/+* mice consumed approximately 15 to 20% more food than wild type littermates (Figure 1C and 1D). There was a significant increase in the percentage of body fat and slightly reduced lean body mass in *Tw/+* mice of both sexes (Table 1), indicating that fat accounts for the increased body mass. Body weight-adjusted energy expenditure, estimated from oxygen consumption, revealed a reduced metabolic rate in *Tw/+* mice that did not reach statistical significance (Table 1). *Tw/+* mice had normal serum glucose levels but elevated levels of serum free fatty acids, triglycerides, insulin, leptin, corticosterone and adiponectin (Table 1). Insulin and glucose tolerance tests of 15-week-old females showed insulin resistance and slight glucose intolerance in *Tw/+* mice (Figure 1E and 1F), consistent with data for other obese mice with hyperinsulinemia [17].

**Table 1:** Metabolic parameters of wild-type and *Tw/+* mice.

	Male		Female	
	+/+	*Tw*/+	+/+	*Tw*/+
Body weight (g)	30.2±0.7	41.2±1.8 *	25.4±0.8	40.1±1.7 *
Lean mass (g)	24.7±0.4	20.8±0.6 *	20.2±0.4	17.9±0.4 *
Fat mass (g)	4.6±0.6	19.7±1.2 *	4.1±1	21.8±1.6 *
Glucose (mg/dl)	128±8	135±27	127±2	106±3 *
Free fatty acids (mM)	0.33±0.06	0.65±0.4 *	0.21±0.03	0.46±0.04 *
Triglycerides (mg/ml)	89±11	145±16 *	58.6±9	137.2±21 *
Insulin (ng/ml)	1.2±0.2	35.5±3.7 *	0.66±0.08	42.5±14 *
Leptin (ng/ml)	11±1	87.2±5.1 *	10.5±3.9	99.3±9.8 *
Corticosterone (ng/ml)	157±14	375±34 *	236±22	566±52 *
Adiponectin (mg/ml)	9.8±1.3	25.8±3.6 *	15.9±2.1	42.6±9.2 *
O_2_ consumption (ml/h)	126±4	158±9 *	128±5	174±8 *
O_2_ consumption (ml/g^0.75^)	10.9±0.6	10.4±0.4	12.6±0.7	11.3±0.3
Motor activity (beam breaks/h)	143±13	49±9 *	230±34	117±53 *

All data represent the mean ± SEM. n = 6 for each sex and genotype group. Lean and fat mass were based on DEXA analysis. Asterisks indicate P<0.05 in an unpaired Student's *t* test.

### Inner ear phenotype of Twirler mice

**Figure 2:**
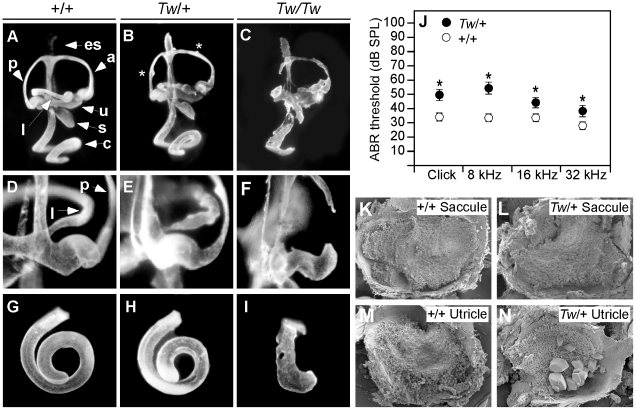
Inner ear morphology, hearing thresholds, and otoconia of Twirler mice. Paint-filled inner ears of wild type, heterozygous and homozygous *Tw* mice at E14.5 are shown. Lateral views (A-C) show the entire cochlea, vestibular labyrinth and endolymphatic sac. Medial views (D-F) show the non-ampullated end of the lateral semicircular canal. Ventral views (G-I) show the cochlear duct. The overall structure of *Tw/+* inner ears was intact, but the contours of the semicircular canals were irregular due to small bulges and projections along the canals (indicated by asterisks in B). There were irregularities and constrictions of the non-ampullated ends of lateral canals (E). Shortened cochlear ducts are consistently observed in *Tw/+* ears (H). *Tw*/*Tw* inner ear anatomy is disrupted but recognizable (C, F, I). *Tw*/*Tw* semicircular canals and cochlear ducts were either discontinuous or ruptured. Average ABR thresholds for all wild type ears (n = 24) are shown as white circles and *Tw/+* ears (n = 24) as black circles with the standard error of the mean (SEM). Results are shown for click, 8-, 16- and 32-kHz pure-tone stimuli (J). Scanning electron microscopy showed no difference in otoconia between wild type (K) and *Tw/+* saccules (L) at P6. *Tw/+* utricles (N) had giant otoconia. a, anterior semicircular canal; c, cochlear duct; es, endolymphatic sac; l, lateral semicircular canal; p, posterior semicircular canal; s, saccule; u, utricle.

We evaluated the morphology of mutant inner ears using the paint-filling technique (Figure 2). The *Tw/+* inner ears had grossly intact semicircular canals and neurosensory cristae ampullaris, but the contours of the canals were irregular due to small bulges and projections (Figure 2B). The most anatomically consistent malformation was found at the non-ampullated end of the lateral canal where it normally narrows to join the vestibule in wild type ears (Figure 2D). In contrast, the non-ampullated ends of *Tw/+* lateral canals were irregular or constricted (Figure 2E). *Tw/Tw* inner ears have more severe malformations that include absence of the lateral semicircular canal, truncation of the posterior semicircular canal, and shortening of the cochlear duct (Figure 2C, 2F and 2I).

The average length of *Tw/+* cochlear ducts (Figure 2H) was 91% (±5%) that of wild type ears (P<0.00002; Figure 2G). Binaural average ABR thresholds were elevated for *Tw/+* mice in comparison to wild type controls at one month of age (33±1.6 dBSPL vs. 55±5.3 dBSPL at 8 kHz, p<0.0006; 33±1.8 dBSPL vs. 46±4 dBSPL at 16 kHz, p<0.01; 29±1.9 dBSPL vs. 39±3.6 dBSPL at 32 kHz, p<0.023; Figure 2J). *Tw/+* mice showed no significant change in ABR thresholds measured at three months of age in comparison to thresholds measured at one month of age (not shown).

*Tw/+* utricles had giant otoconia that were transparent by light microscopic examination but visible by scanning electron microscopy (Figure 2N). In contrast, *Tw/+* saccular otoconia appeared normal (Figure 2L).

### Linkage mapping and positional cloning of *Tw*

**Figure 3:**
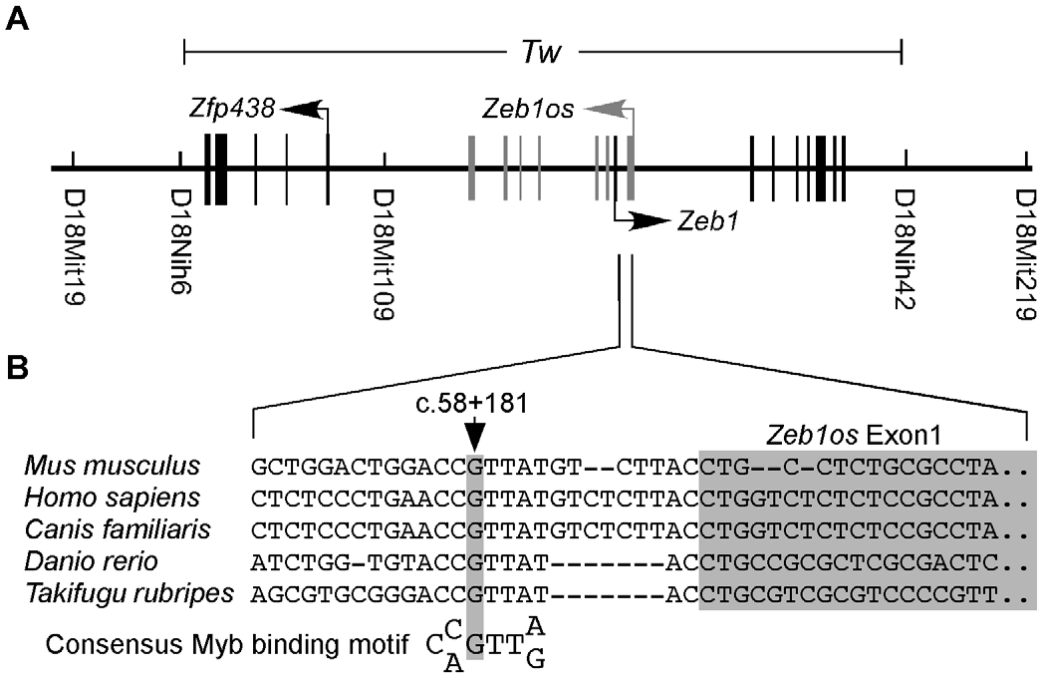
Linkage mapping and identification of *Tw* mutation. (A) Meiotic recombinations in affected mice localized *Tw* to an 814-kb interval between D18nih6 and D18nih42 on proximal chromosome 18. Three genes are located within the *Tw* critical region. A single nucleotide substitution (c.58+181G>A) was detected in the *Tw* allele in the region of overlapping first introns of *Zeb1* and *Zeb1os*. (B) c.58+181G is evolutionarily conserved and c.58+181G>A disrupts a consensus binding sequence for the Myb family of transcription factors.

We screened 1679 [(C57BL/6J-*Tw/+* x CAST/Ei)F1-*Tw/+* x C57BL/6J]N2 progeny for recombinations. Recombination locations were refined with additional markers to narrow the *Tw* interval to 814 kb between D18Nih6 and D18Nih42 (Figure 3A). This interval was five Mb proximal to the Tg9257 transgene insertion site [3]. The *Tw* interval contained three genes: *Zeb1*, *Zeb1os* (*Zeb1* opposite strand transcript, annotated in MGI as predicted gene *Gm10125*) and *Zfp438* (Figure 3A). *Zeb1* encodes a transcription factor with two zinc finger motifs and one homeobox motif. *Zeb1os* is predicted to encode a long noncoding RNA of unknown function. It is located on the opposite strand of *Zeb1* where the two overlapping genes share parts of their first introns. Finally, *Zfp438* is predicted to encode a zinc finger protein whose biological function is unknown [18].

*Zeb1* was a good candidate for the gene mutated in *Tw* based upon the phenotype associated with a targeted deletion allele, *Zeb1^ΔEx1^*. Homozygous *Zeb1^ΔEx1^* mice are born with cleft palate, skeletal and thymus abnormalities, and die shortly after birth [10]. We observed that *Zeb1^ΔEx1/+^* heterozygotes have inner ear morphology and hearing thresholds that are indistinguishable from those of wild type littermates, whereas *Zeb1^ΔEx1/ΔEx1^* homozygotes have a subtle constriction of the midportion of the lateral semicircular canal that differs in location and severity from that observed in *Tw/+* mice (Figure S1). This difference is probably not due to genetic background since both lines were congenic on a C57BL/6J background.

To determine if *Tw* and *Zeb1^ΔEx1^* can complement to form a normal palate or inner ear, we crossed heterozygous *Tw* and heterozygous *Zeb1^ΔEx1^* mice. We observed an approximate Mendelian ratio of genotypes: five +/+, five *Tw*/+, seven *Zeb1^ΔEx1^* ^/+^ and eight *Tw*/*Zeb1^ΔEx1^*. All *Tw*/*Zeb1^ΔEx1^* mice were born with normal palates and developed into adults with circling behavior typical of *Tw/+* mice. The lateral semicircular canals resembled those of *Tw/+* mice (Figure S1). These results suggest these mutations exert their effects via different genes or mechanisms. While the *Zeb1* pathway may be altered in Twirler mice, it is unlikely to be due to a loss-of-function allele of *Zeb1*.

To identify the *Tw* mutation, we first used 5′-RACE and 3′-RACE to identify novel exons of *Zeb1*, *Zeb1os* and *Zfp438.* 5′-RACE revealed *Zeb1* transcripts with each of five additional alternative first exons (designated 1b, 1c, 1d, 1e and 1f) between exon 1 (heretofore termed exon 1a) and exon 2 (Figure S2). We amplified and sequenced all novel and annotated exons of *Zeb1*, *Zeb1os* and *Zfp438* from genomic DNA of *Tw/Tw, Tw/+* and wild type mice. We also amplified and sequenced cDNA transcripts of these genes from embryonic mRNA. All major transcripts of these genes were amplified from mice with each genotype. We found no sequence differences in the cDNAs or genomic exons. Sequence analysis of the 192-bp region of overlap of *Zeb1* and *Zeb1os* revealed a single nucleotide substitution (G>A) 181 bp downstream of *Zeb1* exon 1 and 12 bp downstream of *Zeb1os* exon 1 in *Tw* (Figure 3B). We designated this *Tw* variant as c.58+181G>A, which was the only sequence variation we detected. The wild type variant c.58+181G was conserved among 13 normal control inbred mouse strains as well as other vertebrate species (Figure 3B). *In silico* analyses (NNsplice, GeneSplicer, Net2Gene) predict that c.58+181G>A does not affect splicing of the adjacent splice donor site for exon 1 of *Zeb1os*. Sequence analysis of *Zeb1* and *Zeb1os* cDNA transcripts confirmed no effect of c.58+181G>A on splicing.

### Electrophoretic mobility shift assay of *Tw* DNA

**Figure 4:**
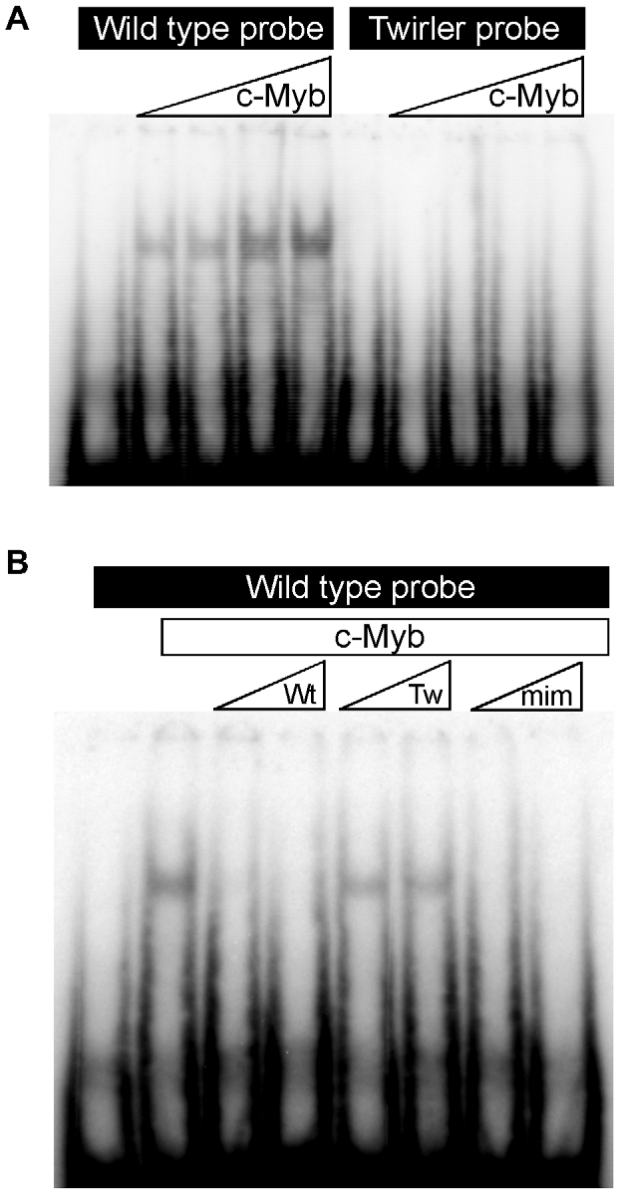
Electrophoretic mobility shift assay of Twirler DNA and C-Myb. Double-stranded oligodeoxyribonucleotide probes encoding wild type or Twirler sequences were incubated with increasing amounts of purified mouse C-Myb. (A) A mobility shift was observed with the wild type probe but not with the Twirler probe. (B) The wild type DNA probe and C-Myb were co-incubated with 25- or 50-fold molar excess of unlabeled wild type, Twirler or mim-1 competitor probes. The shift of labeled wild type probe mobility was completely inhibited by the addition of wild type or mim-1 competitors, but not by the Twirler competitor.

c.58+181G>A disrupts a predicted site for Myb protein binding (Figure 3B)[16]. To test if this change can alter the binding of a Myb protein, recombinant mouse C-Myb was expressed and purified for an electrophoretic mobility shift assay (EMSA) of its binding to oligonucleotide probes containing either c.58+181G or c.58+181A and the flanking genomic sequences. There was a shift of the mobility of the wild type DNA probe in the presence of C-Myb, while the *Tw* DNA probe mobility was unchanged (Figure 4A). The binding of C-Myb to wild type DNA was inhibited by both the wild type probe and a *mim-1* control probe which has been shown to interact with C-Myb [19], but not by the *Tw* probe (Figure 4B). These data provide *in vitro* evidence that the *Tw* mutation can disrupt binding of a Myb protein (C-Myb) to the mutated first intronic sequence of *Zeb1.*

### Quantitative RT-PCR analysis of *Zeb1^Tw^* and *Zeb1^ΔEx1^* transcripts

**Figure 5:**
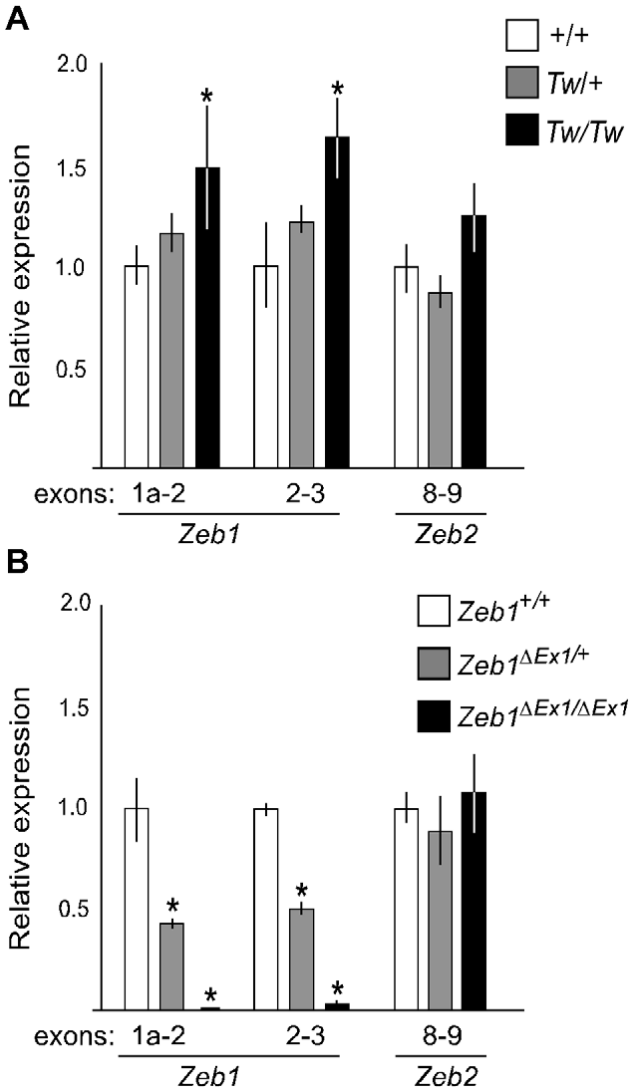
Comparative RT-PCR analysis of *Tw* and *Zeb1^ΔEx1^* RNA from E13.5 ears. (A) Increased *Zeb1* RNA levels were detected in *Tw/*+ and *Tw/Tw* ears compared with those of wild type littermates. (B) In *Zeb1^ΔEx1^* ears, there were no detectable *Zeb1^ΔEx1^* transcripts containing exon 1 (as expected). *Zeb1* transcripts containing exons 2 and 3 were nearly absent in *Zeb1^ΔEx1/ΔEx1^* ears. *Zeb2* RNA levels in *Tw* and *Zeb1^ΔEx1^* ears were unchanged from wild type levels. Asterisks indicate P<0.05.

We analyzed mRNA expression levels of *Zeb1*, *Zeb1os* and *Zfp438* from inner ears of *Tw/Tw, Tw/+* or wild type mice at E13.5. We performed the same analysis with *Zeb1^ΔEx1^* heterozygotes, homozygotes, and wild type littermates. We designed primer pairs to specifically amplify *Zeb1* transcripts starting from each of exons 1a, 1b, 1c, 1d, 1e or 1f. One primer pair for constitutively spliced exons 2 and 3 was designed to amplify all *Zeb1* transcripts. The levels of *Zeb1* transcripts containing exon 1b, 1c, 1d, 1e, or 1f, as well as the *Zeb1os* and *Zfp438* transcripts, were too low to be reliably quantified by RT-PCR. The levels of transcripts containing exons 1a and 2, as well as exons 2 and 3, were significantly increased from the *Tw* allele of *Zeb1* (*Zeb1^Tw^*) in comparison to wild type *Zeb1* (Figure 5A). In contrast, *Zeb1^ΔEx1^* expressed no *Zeb1* transcripts containing exons 1a and 2, and nearly non-detectable levels of any other *Zeb1* transcripts containing other exons (Figure 5B). Transcripts levels for the closely related *Zeb2* gene were unchanged among all three *Zeb1* genotypes (Figure 5B). These results indicate that *Zeb1^ΔEx1^* is a loss-of-function allele whereas *Zeb1^Tw^* is likely to act via gain-of-function.

### A mouse knockin of the *Zeb1^Tw^* intron 1 sequence variant recapitulates the Twirler phenotype

**Figure 6:**
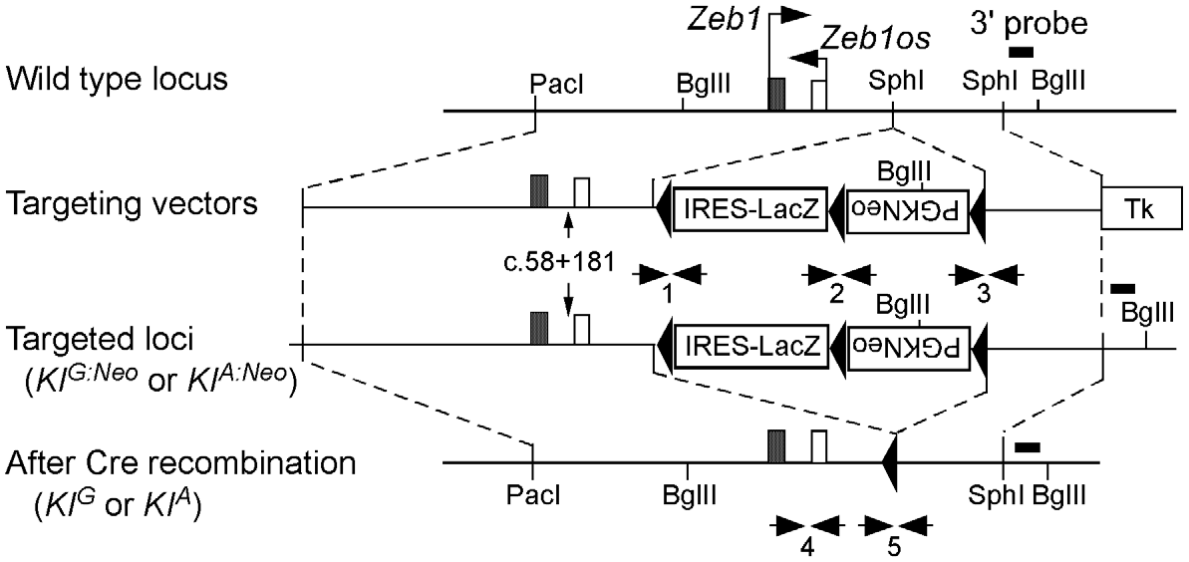
Generation of wild-type (*KI^G^*) and *Tw* (*KI^A^*) knockin mice. Genomic structure, targeting vectors, and targeted locus before (*KI^G:Neo^*, *KI^A:Neo^*) and after (*KI^G^*, *KI^A^*) excision of the *lacZ*-PGK-Neo^R^ cassette by Cre recombinase. Arrows indicate genotyping primer pairs (Table S1). The 3′ Southern blot probe is indicated (Figure S3).

**Figure 7:**
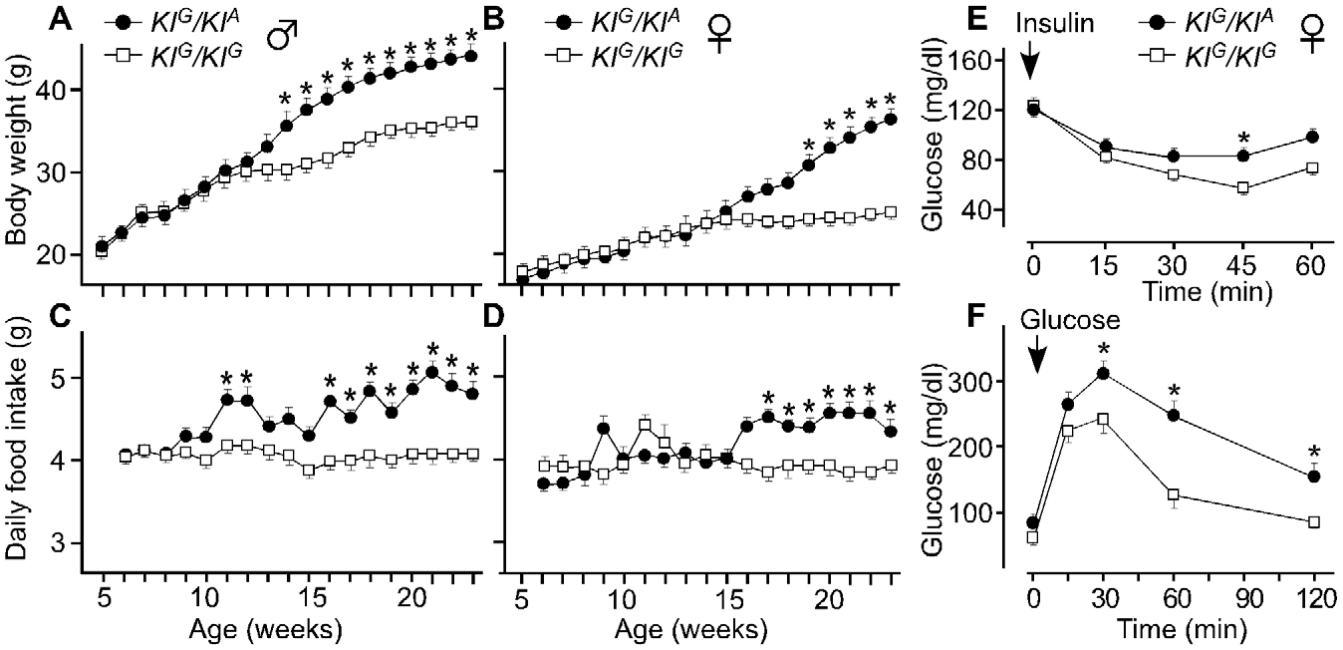
Body weight, daily food intake, and insulin and glucose tolerance of knockin mice. Body weight was measured weekly in males (A) and females (B) (n = 7 for each sex and each genotype). Food intake was measured weekly to calculate average daily intake for males (C) and females (D). *KI^G^/KI^A^* males and females show increased body weight after 14 (p<0.017) and 19 weeks (p<0.018) of age, respectively. Both *KI^G^/KI^A^* males and females show increased food intake after 11 (p<0.02) and 17 weeks (p<0.03) of age, respectively. Serum glucose levels are shown for *KI^G^/KI^A^* (n = 7) and *KI^G^/KI^G^* (n = 7) female mice injected with insulin (E) or glucose (F) at 25 weeks of age. Mean body weights were 36.8 g and 25.4 g for *KI^G^/KI^A^* and *KI^G^/KI^G^* mice, respectively. *KI^G^/KI^A^* mice show insulin resistance. In the glucose tolerance test, the area under the curve (AUC) is significantly higher in *KI^A^/KI^G^* compared with *KI^G^/KI^G^* mice (p = 0.004). The vertical bars indicate the standard error of the mean. Asterisks indicate P<0.05 in an unpaired Student's *t* test.

**Figure 8:**
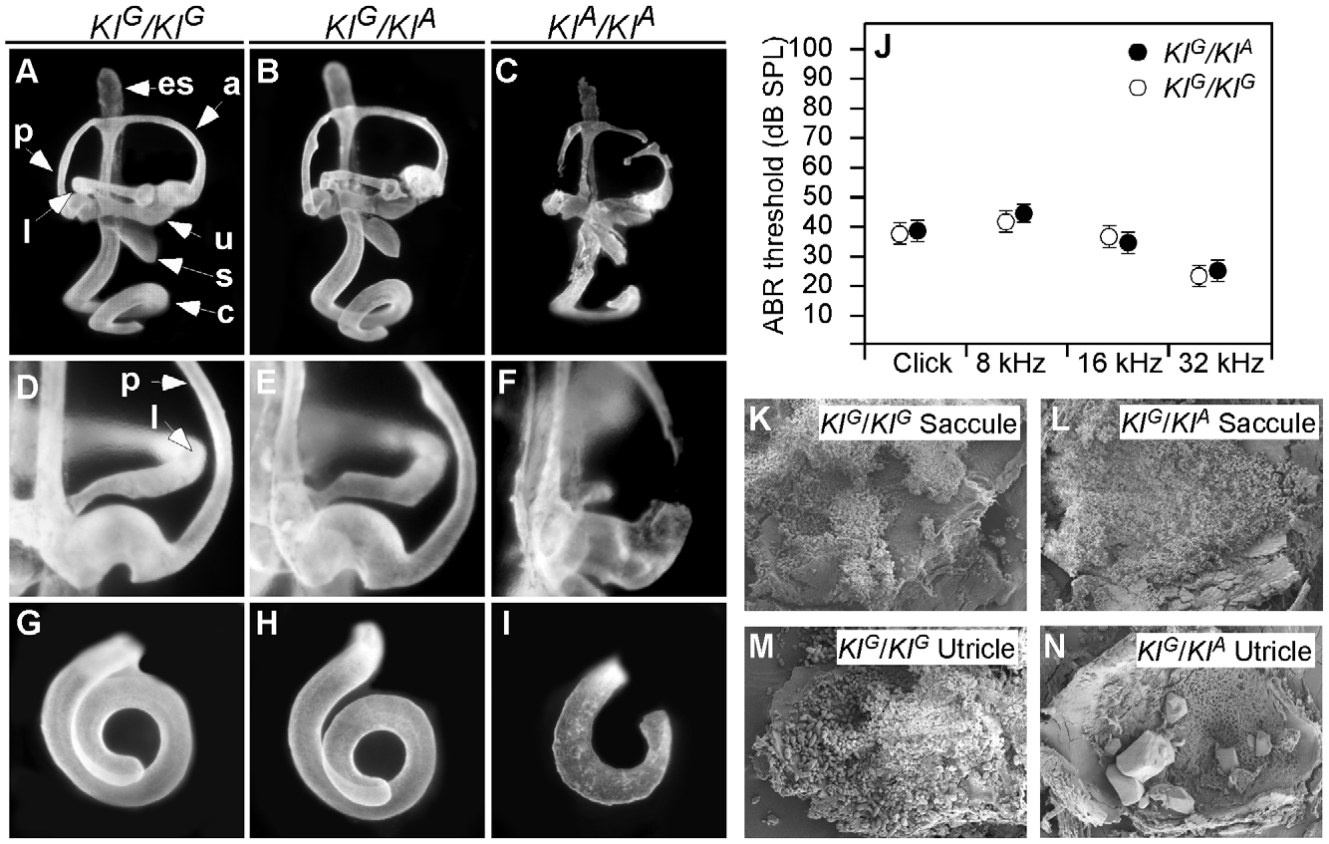
Inner ear morphology, hearing thresholds, and otoconia of wild-type (*KI^G^*) and *Tw* (*KI^A^*) knockin mice. Paint-filled inner ears of *KI^G^/KI^G^*, *KI^G^/KI^A^* and *KI^A^/KI^A^* mice at E14.5 are shown from lateral (A-C), medial (D-F) and ventral (G-I) views. *KI^G^/KI^G^* inner ear morphology appeared normal. *KI^G^/KI^A^* ears had irregular contours of the semicircular canals (B), but we did not observe abnormal constrictions at the non-ampullated ends of the lateral semicircular canals (E). *KI^A^/KI^A^* inner ear structure is very abnormal (C), with discontinuous or partially ruptured semicircular canals (F) and shorter cochlear ducts (I). Average ABR thresholds for all *KI^G^/KI^G^* ears (n = 10) are shown as white circles and *KI^G^/KI^A^* ears (n = 26) as black circles with the standard error of the mean (SEM). Scanning electron microscopy showed no difference in otoconia between *KI^G^/KI^G^* (K) and *KI^G^/KI^A^* saccules (L) at P6. *KI^G^/KI^A^* utricles (N) had giant otoconia. a, anterior semicircular canal; c, cochlear duct; es, endolymphatic sac; l, lateral semicircular canal; p, posterior semicircular canal; s, saccule; u, utricle.

**Figure 9:**
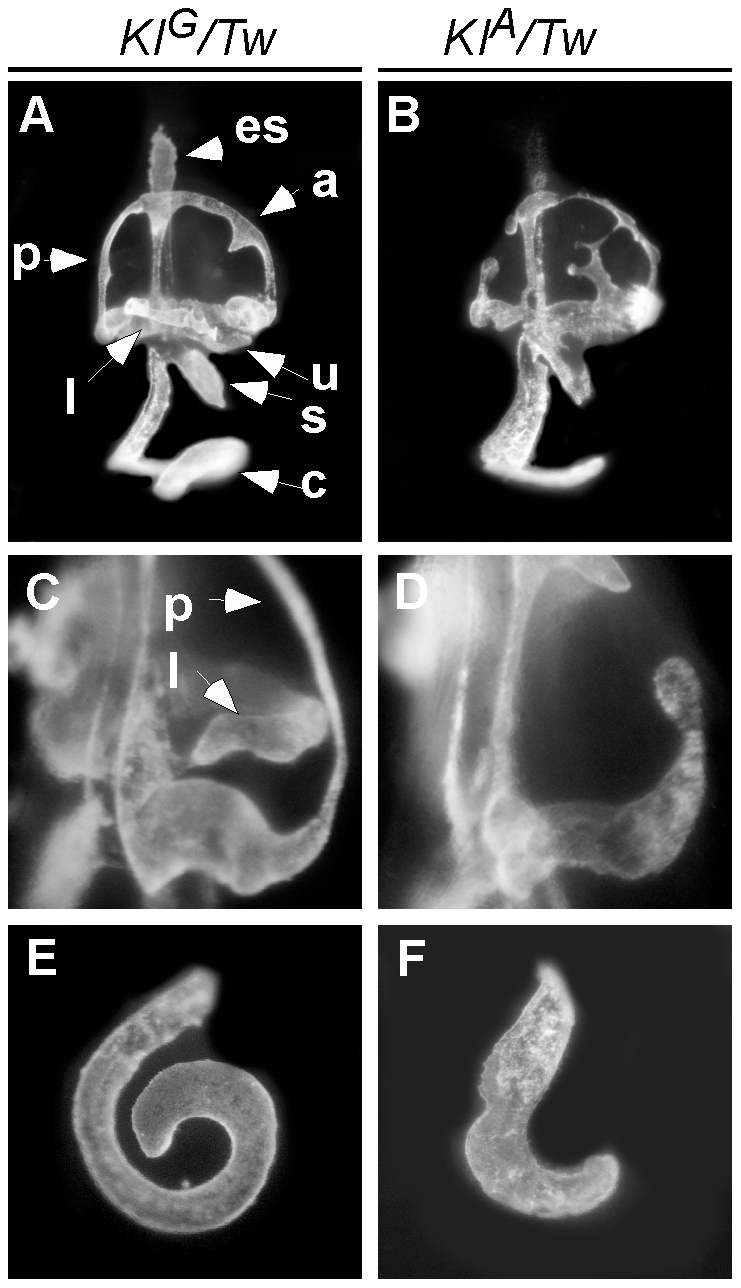
Compound heterozygous *KI^G^*/*Tw* and *KI^A^/Tw* inner ear morphology. Paint-filled inner ears of *KI^G^/Tw* and *KI^A^/Tw* mice at E14.5 are shown from lateral (A and B), medial (C and D) and ventral (E and F) views. In *KI^G^/Tw* ears, the irregular contours of the semicircular canals, the constriction of the non-ampullated end of the lateral semicircular canal, and the short cochlear duct were all indistinguishable from similar findings in *Tw/+* ears (see Figure 2). In *KI^A^/Tw* ears, the discontinuous or ruptured semicircular canals and cochlear ducts were indistinguishable from those of *Tw/Tw* (Figure 2) and *KI^A^/KI^A^* ears (Figure 8). a, anterior semicircular canal; c, cochlea duct; es, endolymphatic sac; l, lateral semicircular canal; p, posterior semicircular canal; s, saccule; u, utricle.

To confirm the pathogenic effect of c.58+181G>A, we generated two knockin mouse lines: *KI^A^* segregates the *Tw* variant c.58+181A and *KI^G^* segregates the wild type variant c.58+181G (Figure 6). Compound heterozygous *KI^G^/KI^A^* mice consumed more food and grew heavier with increased adiposity in comparison to *KI^G^/KI^G^* control males and females (Figure 7A–7D, Table 2). The energy expenditure and circulating hormone levels in *KI^G^/KI^A^* mice recapitulated the *Tw/+* phenotype (Table 2). The reduction in body weight-adjusted energy expenditure reached statistical significance in *KI^G^/KI^A^* female mice, whereas it did not in *Tw/+* females (Table 1). Insulin and glucose tolerance tests showed insulin resistance and slight glucose intolerance in *KI^G^/KI^A^* mice (Figure 7E and 7F). Although *KI^G^/KI^A^* mice showed neither circling behavior nor constricted semicircular canals, the semicircular canals were irregular (Figure 8B) and the utricles contained giant otoconia (Figure 8N). Average ABR thresholds for *KI^G^/KI^A^* and *KI^G^/KI^G^* mice were not significantly different (Figure 8J). *KI^A^/KI^A^* and *KI^A^/Tw* inner ears displayed the same malformations as *Tw/Tw* ears (Figure 8C, 8F, 8I, and Figure 9B, 9D, 9F). *KI^G^/KI^A^* average spleen weight was decreased by 15% (P<0.05) but average thymus weight did not differ relative to *KI^G^/KI^G^* littermates (Table 2). We observed cleft palate with or without cleft lip in *KI^A^/KI^A^* and *KI^A^/Tw* mice with 50% and 90% penetrance, respectively (not shown). We did not observe cleft palate or cleft lip in *KI^G^/KI^A^*, *KI^G^/KI^G^* or *KI^G^/Tw* mice, indicating that the recapitulation of the *Tw* phenotype is specific.

**Table 2:** Metabolic parameters of knockin mice.

	Male		Female	
	*KI^G^*/*KI^G^*	*KI^A^*/*KI^G^*	*KI^G^*/*KI^G^*	*KI^A^*/*KI^G^*
Body weight (g)	36.4±1.1	45.2±1.6 *	25.2±0.8	36.7±2.9 *
Lean mass (g)	26.6±0.5	24.9±0.6 *	20.4±0.4	19.5±0.4
Fat mass (g)	8.6±0.8	19.0±1.6 *	3.6±0.6	16.0±2.7 *
Glucose (mg/dl)	124±7	126±24	110±5	94±2 *
Free fatty acids (mM)	0.51±0.03	0.56±0.04	0.25±0.04	0.46±0.04 *
Triglycerides (mg/ml)	115±10	199±13 *	67.2±7.1	147±15 *
Insulin (ng/ml)	1.2±0.1	8.9±1.1 *	0.47±0.04	4.5±1.5 *
Leptin (ng/ml)	4.6±0.6	9.1±1.3 *	6.2±0.5	19.9±2.4 *
Corticosterone (ng/ml)	99±20	226±20 *	189±18	301±40 *
Adiponectin (mg/ml)	4.6±0.6	9.1±3.6 *	6.2±0.5	19.9±2.4 *
O_2_ consumption (ml/h)	115±3	134±6.8 *	106±2	123±6 *
O_2_ consumption (ml/g^0.75^)	7.4±0.1	7.5±0.2	9.3±0.1	7.9±0.2 *
Motor activity (beam breaks/h)	115±19	77±14 *	216±34	104±12 *

All data represent the mean±SEM. n = 7 for each sex and genotype group. Lean and fat mass were based on DEXA analysis. Asterisks indicate P<0.05 in an unpaired Student's *t* test.

The different phenotypic severity and penetrance of *KI^A^* in comparison to *Tw* could result from genetic background differences, since *Tw* arose on a different undefined stock. However, we serially backcrossed *Tw* to wild type C57BL/6J for over 30 generations, and *KI^A^* was generated from C57BL/6-derived Bruce4 ES cells and maintained on an isogenic C57BL/6J background. Therefore the differences in severity and penetrance could result from closely linked sequence variation, the residual loxP site in *KI^A^*, or a combination of these effects.

### Zeb1 protein expression in Twirler inner ears

**Figure 10:**
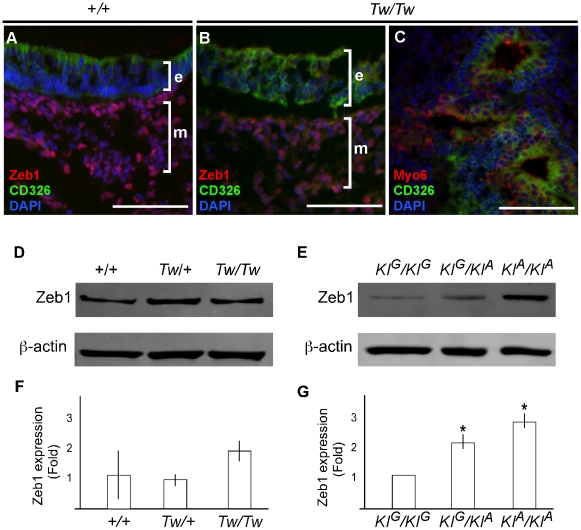
Zeb1 protein expression in *Tw/Tw* inner ears. Vestibular tissue from wild type (A) or *Tw/Tw* (B, C) mice at P0 was stained with antibodies against Zeb1 (A and B), the sensory hair cell marker Myo6 (C), the epithelial cell marker CD326 (A, B, C), or DAPI to label cell nuclei (A, B, C). Scale bar  =  100 µm. Zeb1 was detected at similar levels in wild type and *Tw/Tw* mesenchymal cells. e, epithelial layer; c, mesenchymal layer. Inner ear structures are difficult to identify with disrupted mesenchymal- and epithelial-specific microarchitecture and gene expression in some *Tw/Tw* ears (C). (D–G) Representative western blot analyses of E13.5 mouse-head protein extracts from individual *Tw* (D) and knockin animals (E) and littermate controls. Each Zeb1 band intensity measurement was normalized to the β-actin band intensity for that sample. These Zeb1/β-actin band intensity ratios for *Tw* samples were then normalized to mean wild type (*+/+*) ratios calculated from the same gel. Mean normalized Zeb1 expression values are shown for +/+ (n = 3), *Tw*/+ (n = 2) or *Tw/Tw* (n = 2) mice (F) and *KI^G^/KI^G^* (n = 2), *KI^G^/KI^A^* (n = 2) or *KI^A^/KI^A^* (n = 6) mice (G). The indicated variation in the *+/+* mean value in (A) but not (B) reflects the inclusion of two *+/+* samples on one of the gels for the *Tw* (A) analysis but only one *+/+* sample on each gel for the knockin analysis (B). ANOVA analysis revealed that the observed differences among +/+, *Tw*/+ and *Tw/Tw* mice were not significant (P>0.05), but the differences among *KI^G^/KI^G^*, *KI^G^/KI^A^* and *KI^A^/KI^A^* mice were significant (P<0.05).

To determine if Zeb1 protein is expressed from the *Tw* allele, we stained inner ears of *Tw/Tw* mice with anti-Zeb1 antibodies (Figure 10A and 10B). We observed Zeb1 expression in non-epithelial (mesenchymal) cells surrounding *Tw/Tw* inner ears in which epithelial and mesenchymal tissue compartments could be microanatomically differentiated (Figure 10B). Other *Tw/Tw* inner ears had poorly preserved microarchitecture, precluding a differentiation of epithelium versus mesenchyme (Figure 10C). We conclude that Zeb1 protein is expressed in *Tw/Tw* ears, consistent with the result of real-time RT-PCR.

To determine if Zeb1 protein levels are altered by *Tw*, we performed a western blot analysis of inner-ear or whole-head protein extracts from E13.5 mice. We compared *Tw/Tw, Tw*/+ and wild type littermates, as well as *KI^G^/KI^G^*, *KI^G^/KI^A^* and *KI^A^/KI^A^* littermates. We were unable to detect Zeb1 in inner-ear protein extracts, but were able to reliably detect it in samples from whole heads. Total Zeb1 protein levels appeared to be slightly increased by *Tw* in comparison to wild type littermates (Figure 10D). This difference was not significant (ANOVA, P>0.05), possibly due to small numbers of animals and the degree of variation of Zeb1 band intensities within genotypes (Figure 10F). In contrast, Zeb1 protein levels in *KI^G^/KI^A^* and *KI^A^/KI^A^* mice were 2- to 3-fold higher than in *KI^G^/KI^G^* littermates (Figure 10E). The variation within knockin genotype groups was smaller, resulting in differences between knockin genotype groups that were significant (P<0.05) (Figure 10G).

## Discussion

This study demonstrates that the phenotype of Twirler is caused by a noncoding nucleotide substitution within a shared first intron of the *Zeb1* and *Zeb1os* genes on mouse chromosome 18. This is a rare example of a Mendelian noncoding point mutation that does not affect a splice site or promoter. Our results demonstrate the potential for complex phenotypic effects of noncoding point variants, which are increasingly implicated in association studies of genetically complex traits. Our recombinant knockin mouse model and wild type knockin control for testing the pathogenic potential of the *Tw* mutation may be a useful paradigm to explore the effects of other noncoding variants of unknown pathogenic potential. The altered penetrance potentially associated with a residual loxP site in the *Tw* knockin line serves a cautionary note to include a wild type knockin control.

Although the initial study by Lyon [1] described abnormal development of the sensory neuroepithelium in the cristae ampullaris of some semicircular canals of *Tw/+* mice, we have not observed the same alteration. Instead we observed a highly penetrant constriction of the non-ampullated end of the lateral semicircular canal that could impede or prevent the flow of endolymph and disrupt neurosensory detection of angular acceleration. A difference in strain background [1] may account for the different result. Moreover, Lyon reported that utricular otoconia were absent in *Tw/+* ears whereas we observed giant utricular otoconia. This difference could also result from the strain background difference, loss of giant otoconia during the dissection process, or our use of scanning electron microscopy in addition to light microscopy. Nevertheless, either of the described utricular phenotypes could impair the detection of linear acceleration by *Tw/+* utricles. We conclude that our observed semicircular canal and utricular anomalies underlie the vestibular behaviors of *Tw/+* mice, although we cannot estimate their relative contributions to the observed vestibular functional phenotype. Correlating mouse vestibular structural or functional abnormalities with behavior is difficult due to a complex interrelationship between vestibular behavior and anxiety that is also dependent upon strain background [20].

The cause of hearing loss observed in some *Tw/+* mice also remains obscure. Postmortem examination of middle ears did not reveal otitis media or developmental malformations of the external or middle ears that could account for the hearing loss. Although severe hearing loss has been observed in other mouse mutants with much shorter cochlear ducts [21], the severity of hearing loss in *Tw/+* mice was highly variable but the degree of shortening of the cochlear duct was nearly constant. This lack of correlation leads us to conclude that associated physiologic defects or undetected structural anomalies underlie hearing loss in *Tw/+* mice.

The *Tw/+* phenotype includes hyperphagia with elevated levels of circulating corticosterone and adiponectin that are similar to those in a corticotropin-releasing factor (CRF) transgenic mouse model of Cushing syndrome [22], [23]. Other phenotypic similarities of that model to *Tw/+* include increased body weight and adiposity, alopecia, atrophy of the thymus and spleen, and muscle wasting. This may suggest that *Tw* disrupts, at least in part, the hypothalamus-pituitary-adrenal axis. This is consistent with expression of *Zeb1* in the pituitary gland [24]. However, Zeb1 protein is also expressed in adipose tissue and increases during adipogenesis in cell culture [11]. Moreover, *Zeb1^ΔEx1/+^* mice develop obesity that is not associated with hyperphagia [11], unlike *Tw/+* mice (Figure 1C and 1D). Therefore different mechanisms or tissues may underlie obesity phenotypes associated with *Zeb1^ΔEx1^* and *Zeb1^Tw^*. The pathogenetic mechanism for one or both of these *Zeb1* alleles may also underlie a locus for susceptibility to obesity on human chromosome 10p11 [25]-[28], which includes the human *ZEB1* gene.

The results presented here and in Hertzano et al. [13], in combination with the body of published data on *Zeb1* in cancer and normal development, show that *Zeb1* is a master regulator of mesenchyme-specific gene expression in the developing mouse ear. Twirler is a novel example of a disorder of hearing or balance caused by a disruption of mesenchymal-epithelial identities or interactions. A similar lateral semicircular canal phenotype is seen in other hyperactive circling mice, including *epistatic circler* mice [29] and mice segregating a gene-trap allele of *Chd7* [30]. *Chd7* encodes a chromodomain protein required for the development of multipotent migratory neural crest cells [31], which includes an epithelial-to-mesenchymal transition. An auditory-vestibular phenotype approximating that of Twirler and *Chd7* mutant mice is also observed in human patients with CHARGE syndrome and mutations of the human *CHD7* gene [32], [33]. Semicircular canal formation is also known to require Bmp4 [34] and heterozygosity for a knockout allele of mouse *Bmp4* primarily affects the lateral semicircular canal [35]. Bmp4 is a member of the transforming growth factor-β (TGF-β) super-family [36], providing another link to Zeb1 since Zeb1 and Zeb2 have been implicated in TGF-beta/BMP signaling [8].

Why do Twirler mice have a different inner ear phenotype than *Zeb1^ΔEx1^* mice? Genetic background differences seem unlikely to account for this difference since *Zeb1^ΔEx1^* and Twirler were both maintained on a congenic C57BL/6J background. It is possible that other *Zeb1* transcripts could compensate for the loss of exon 1 in *Zeb1^ΔEx1^* ears, but our quantitative RT-PCR and expression profiling results [13] render this hypothesis unlikely. Alternatively, the closely related *Zeb2* gene may be able to compensate for the loss of *Zeb1* expression in the inner ear, but not other affected tissues such as the palate or lymphoid system. However, our quantitative RT-PCR results revealed no compensatory change in *Zeb2* transcript levels in the mutants. It is also possible that disruption of *Zeb1os* may contribute to the *Tw* phenotype. Ectopic expression of an analogous long noncoding antisense RNA in epithelial cells leads to altered *Zeb2* RNA splicing, increased Zeb2 protein levels, and epithelial-to-mesenchymal transition [37]. However, *Zeb1os* RNA levels were too low for us to reliably detect and monitor by either qRT-PCR or Northern blot analyses to confidently address this possibility (data not shown). Finally, perhaps Twirler does not exert its pathogenic effect via Zeb1. This also seems unlikely since there is significant phenotypic overlap of Twirler with *Zeb1^ΔEx1^*, including abnormalities of the semicircular canals associated with both mutant alleles. Furthermore, the phenotypic effects of compound heterozygosity for *Zeb1^ΔEx1^* and *Tw* are consistent with the conclusion that *Zeb1^ΔEx1^* is an amorphic or hypomorphic allele whereas Twirler acts as a hypermorphic or neomorphic allele to misregulate Zeb1 expression.

Our electrophoretic mobility shift experiment (Figure 4) suggests that *Tw* exerts its pathogenic effect by disruption of binding of C-Myb or other Myb proteins to the first intron of *Zeb1*. There are also published observations supporting the general hypothesis that loss of Myb protein binding leads to de-repression of *Zeb1^Tw^* and inner ear malformations: First, C-Myb can function as either an activator or repressor of gene transcription [38] and is thought to function in regulation of epithelial-mesenchymal cell identity [39]. Second, a pathogenic effect of up-regulation of developmental transcription factors has been demonstrated for *Pax6* in the eye [40] and *Tbx1* in the inner ear [41]. In the inner ear, increased expression of *Tbx1* can cause malformations that include incomplete coiling and reduced extension of the cochlear duct [41]. Furthermore, *Tbx1* expression in the periotic mesenchyme is required for cochlear duct outgrowth [42], suggesting a potential link to the observed inner ear phenotype of Twirler. Taken together, these observations and our results support the hypothesis that Twirler disrupts inner ear development via mis-regulation of *Zeb1*.

The cell type-specific gene expression profiles of Twirler ears [13] suggest that a pathologic disruption of epithelial and mesenchymal cell identities underlies the inner ear malformations. This could arise from a loss of mesenchymal cell identity leading to mesenchymal-epithelial transition (MET), a loss of epithelial cell identity leading to epithelial-mesenchymal transition (EMT), or a combination of these mechanisms. Although the gene expression profiles [13] seem consistent with MET, it is difficult to conceive a simple MET pathway that does not invoke a loss-of-function mechanism in *Tw* mesenchyme. In contrast, EMT would involve a gain-of-function with ectopic expression of Zeb1 in *Tw* inner ear epithelium. Indeed, ectopic expression of Zeb proteins in other epithelial tissues has been shown to lead to EMT in other neoplastic and developmental processes [43]. Distinguishing among EMT and MET mechanisms may be difficult if they involve complex regulatory pathways mediated by *Zeb1os*, *Zeb2*, microRNAs or other genes.

In summary, we have identified the pathogenic mutation of Twirler as a noncoding point mutation that leads to over- or mis-expression of *Zeb1*, pathologic alterations of gene expression [13], cell fate and interactions in the developing inner ear. The ultimate result is a gross alteration of the structure and function of the vestibular and auditory organs. Disruption of epithelial-mesenchymal identity or interactions may be a shared pathogenetic mechanism underlying phenotypes that primarily affect development of the lateral semicircular canals, extension of the cochlear duct, or both.

## Materials and Methods

### Animals

Mice were maintained on a 12∶12-h light-dark cycle. All experiments and procedures were approved by the Animal Care and Use Committees of the National Institute of Diabetes and Digestive and Kidney Diseases, National Institute of Neurological Disorders and Stroke and National Institute on Deafness and Other Communication Disorders. Twirler mice were a kind gift from Drs. Miriam Meisler and Siew-Ging Gong at the University of Michigan and were maintained on a C57BL/6J background by backcrossing heterozygous *Tw* males to C57BL/6J females for at least 30 generations. *Zeb1^ΔEx1^* mice [10] were a generous gift from Dr. Douglas Darling and were serially backcrossed to C57BL/6J to maintain the line.

### Generation of knockin mice

Bacterial artificial chromosome (BAC) clone RP23-135A18 containing mouse genomic DNA encoding exon 1 of *Zeb1* was digested with PacI/SphI and SphI to yield 7.6-kb and 2.6-kb homology arms, respectively, for targeting constructs (Figure 6). Each targeting construct included loxP sites flanking a splice acceptor site and internal ribosomal entry site (IRES) (pGT1.8IresBgeo, provided by Austin Smith at University of Edinburgh) [44], *E. coli lacZ*, and a reverse-oriented pPGK-neomycin resistance cassette cloned into the pPNT plasmid [45] (Figure 6). The wild type (*KI^G^*) and Twirler (*KI^A^*) 7.6-kb PacI/SphI homology arms contained G and A at position c.58+181, respectively. Bruce4 embryonic stem (ES) cells [46] were electroporated with the *KI^G^* or *KI^A^* targeting constructs and grown in the presence of G418 and ganciclovir, using standard protocols at the University of Michigan Transgenic Animal Model Core [47]. G418-resistant ES clones were screened for homologous recombination by PCR and Southern blot analyses. At least three recombinant ES cell lines for each targeting construct were injected into C57BL/6 blastocysts. Chimeric males were mated with C57BL/6 females and offspring were analyzed by Southern blot and PCR analyses for germline transmission of *KI^G^* or *KI^A^* (Figure S3). Table S1 shows PCR primer pairs used to genotype KI alleles before and after neomycin cassette removal (Figure 6). Mice transmitting *KI^G^* or *KI^A^* in the germline were crossed to *Cre* recombinase-expressing mice (C57BL/6-TgN(Zp3-Cre)93Knw, Jackson laboratory, ME) to delete the IRES-*lacZ*-neomycin resistance cassette, leaving a single loxP site 606 bp downstream from *Zeb1* exon 1.

### Phenotype survey

A comprehensive gross anatomical, histological, and serological analysis of three 15-week-old *Tw*/+ and three wild type littermate males was performed as described [48]. Tissue sections from two *Tw/Tw*, two *Tw*/+ and two wild type mice at postnatal day 0 (P0) were analyzed.

### Inner ear phenotype analyses

Heterozygous *Tw* males and females were mated. Pregnant females were identified by the presence of a vaginal plug and gestational stage was estimated by defining that morning as 0.5 days post-conception (dpc). Embryos at 14.5 dpc were harvested and processed for paint-filling as described [49]. The length of the cochlear duct was measured along its outer contour from a ventral view [50]. For scanning electron microscopy (SEM), whole-mounted inner ears were fixed in 2.5% glutaraldehyde in 0.1 M sodium cacodylate with 2 mM CaCl_2_ for 90 min. The organ of Corti, saccule, utricle, and crista ampullaris were dissected free in water and dehydrated with a serial dilution series of acetone. Samples were critical point-dried and sputter-coated followed by visualization with a field-emission scanning electron microscope (S-4800, Hitachi). Auditory brainstem response (ABR) thresholds were measured in response to click or pure-tone stimuli of 8, 16, or 32 kHz as described [51].

### Obesity and metabolic phenotype analyses

Six *Tw/+* male, six *Tw/+* female, six wild type male and six wild type female mice were housed individually with regular mouse chow and water provided *ad libitum*. Body weights were measured weekly from 5 weeks of age. Weekly food intake was measured from weeks 6 through 22 to calculate average daily food intake. At 23 weeks of age, mice were transferred to the NIDDK Mouse Metabolism Core Laboratory for measurement of oxygen consumption, carbon dioxide production and motor activity as described [52]. Body composition was measured using Echo3-in-1 NMR analyzer (Echo Medical Systems, Houston, TX). Tail vein blood was used for serologic analyses. Fifteen-week-old female mice (eight *Tw/+*, six wild type) were tested for glucose and insulin tolerance as described [52]. All data are expressed as a mean ± SEM. Student's *t*-test was used to identify statistically significant differences between genotype groups.

### Linkage backcross

Twirler males (C57BL/6J-*Tw/+)* were crossed with DBA/2J or Castaneus (CAST/Ei) females since Twirler females are poor caretakers of offspring. Male (C57BL/6J-*Tw/+* x DBA/2J)F1-*Tw/+* or (C57BL/6J-*Tw/+* x CAST/Ei)F1-*Tw/+* progeny were backcrossed with DBA/2J or C57BL/6J females, respectively, to generate 337 and 1679 N2 backcross progeny, respectively. Progeny were scored for circling behavior or obesity by visual inspection.

### Recombination mapping

We genotyped short tandem repeat (STR) markers on 337 DBA/2J N2 backcross progeny to identify two STR markers (D18Mit65, D18Mit64, D18Mit19 and D18Umi1) flanking each side of *Tw*. These markers were genotyped in the 1679 CAST/Ei N2 backcross progeny to identify recombinations in the *Tw* region. The *Tw* map interval was defined by genotypes of additional markers in the recombinants. We genotyped MIT markers between D18Mit65 and D18Umi1, as well as 40 novel STR markers (denoted D18Nih1 through D18Nih44; PCR primer sequences listed in Table S1) located between D18Mit19 and D18Mit219.

### Mutation analyses

Genomic DNA of *Tw/Tw, Tw/+* and wild type mice were isolated for PCR amplification as described [53]. The primers were designed to amplify and sequence all of the annotated exons of the *Zeb1, Zeb1os* (MGI predicted gene *Gm10125*) and *Zfp438* genes in the *Tw* critical interval. Additional novel exons were identified by 5′ and 3′- RACE (5′ and 3′ rapid amplification of cDNA ends) of the *Zeb1, Zeb1os* and *Zfp438* genes. This revealed multiple alternative first exons for *Zeb1* that were also sequenced. Reverse transcription (RT)-PCR was performed to amplify and sequence full-length cDNA clones of the three genes using whole body mRNA collected from embryonic *Tw/Tw, Tw/+* and wild type littermates.

PCR reaction conditions were modified to amplify and sequence the overlapping genomic region of *Zeb1* and *Zeb1os*. Fifty-µl PCR reactions contained 50 to 100 ng of genomic DNA, 5 pmol each of forward and reverse primers, 200 mM each dNTP, 0.5 M betaine, 10% dimethyl sulfoxide (DMSO), 2.5 mM MgCl_2_, and 0.5 U of thermostable polymerase. Thermal cycling conditions were: 95°C for 1 min; 33 cycles of 20 s at 95°C, 20 s at 57°C, and 45 s at 72°C; and a final 2-min extension at 72°C. For sequencing, 50 µl PCR reaction products were purified with a QIAquick PCR purification kit (Qiagen, Hilden, Germany) and eluted with 30 µl elution buffer. Three µl of purified products were added to a 10-µl sequencing reaction containing 3.2 pmol primer, 0.25 µl Big Dye Terminator Ready Reaction mix (PE Biosystems), sequencing buffer and 10% DMSO. Cycling conditions were 96°C for 2 min and 33 cycles of 96°C for 10 s, 55°C for 10 s, and 60°C for 4 min. We also amplified and sequenced the overlapping genomic region of *Zeb1* and *Zeb1os* from normal mouse control strains 129/J, AKR/J, BALB/cJ, C3H/HeJ, C57BL/6J, C58/J, CBA/J, CE/J, DBA/2J, P/J, RF/J, SEA/GnJ and SWR/J DNA.

### Electrophoretic mobility shift assay

Double-stranded oligodeoxyribonucleotide probes were synthesized to encode genomic sequences containing c.58+181G (5′-TGCTGGACTGGACCGTTATGTCTTACCTGC and 5′-GCAGGTAAGACATAACGGTCCAGTCCAGCA), c.58+181A (5′-TGCTGGACTGGACCATTATGTCTTACCTGC and 5′-GCAGGTAAGACATAATGGTCCAGTCCAGCA), or a C-Myb binding site control from the *mim-1* gene [19] (5′-GCTCTAAAAAACCGTTATAATGTACAGATATCTT and 5′-AAGATATCTGTACATTATAACGGTTTTTTAGAG). Probes were end-labeled with [γ-^32^P]ATP by T4 Polynucleotide Kinase (New England Biolabs). Mouse C-Myb cDNA was cloned in pET-41a(+) (Novagen), and the protein was expressed in E.*coli* strain BL21(DE3)pLys (Invitrogen) and purified with Ni-NTA columns (Qiagen). Twenty-μl reactions were performed with the EMSA Accessory Kit (Novagen). Unlabeled oligonucleotide competitors were added at 25- or 50-fold molar excess. Binding reaction products were separated by 6% DNA retardation gel electrophoresis (Invitrogen) and visualized with a Typhoon Trio+ (GE Healthcare).

### Quantitative RT-PCR analyses

Inner ears with adjacent mesenchyme were microdissected from E13.5 offspring of *Tw/+* x *Tw/+* matings. Total RNA was isolated from inner ears using PicoPure (Applied Biosystems, Foster City, CA). Total RNA from 10 to 14 ears of the same genotype was pooled and purified with the RNAeasy MinElute Cleanup kit (Qiagen). RNA integrity was measured with an Agilent 2100 Bioanalyzer (Applied Biosystems). One µg of total RNA was reverse-transcribed with oligo(dT) primers and SuperScriptIII (Invitrogen, Carlsbad, CA, USA). For TaqMan real-time PCR, PCR primers were designed to amplify *Zeb1* exons 1a to 2, 1b to 2, 1c to 2, 1d to 2, 1e to 2, 1f to 2, and 2 to 3, *Zeb1os* exons 1 to 2, and *Zfp438* exons 3 to 4 with ZEN double-quenched probes containing a 5′ FAM fluorophore, 3′ IBFQ quencher, and an internal ZEN quencher (IDT, Coralville, IA). Sequences for the primers and probes are listed in Table S1.

Comparative TaqMan assays were performed in triplicate on an ABI 7500 real-time PCR system (Applied Biosystems). PCR reactions were performed in a 50-µl volume containing 5 µl cDNA, 5 µl primer mix (IDT), and 25 µl of Universal PCR Master Mix (Applied Biosystems). Cycling conditions were 50°C for 2 min, 95°C for 10 min, followed by 40 cycles of 15 s at 95°C and 1 min at 60°C. Relative expression was normalized as the percentage of β-actin expression, and calculated using the comparative threshold cycle method of 2^−ΔΔCT^. Data are presented as mean values ± S.D. from six technical replicates. ANOVA was used to identify statistically significant differences between genotype groups (P<0.05).

### Western blot analyses

Proteins were extracted from E13.5 mouse inner ears or whole heads with NE-PER Nuclear and Cytoplasmic Extraction Reagents (Pierce Biotechnology) in the presence of Halt Protease Inhibitor Cocktail (Thermo Fisher Scientific Inc.). Proteins were separated by SDS-PAGE in 4–20% NuPage Bis-Tris gels followed by transfer to PVDF membranes (Millipore Corp., Billerica, MA). Proteins were detected with primary antibodies for Zeb1 (ab64098, Abcam, 1∶200) and β-actin (A2228, Sigma-Aldrich, 1∶1000). Secondary antibodies were conjugated with Cy 3 or Cy 5 (GE Healthcare) and detected with a Typhoon Trio+ (GE Healthcare). Band density was measured using ImageQuant TL software. β-actin levels were used for normalization. ANOVA analysis of two to six biological replicates from each genotype group was used to identify statistically significant differences (P<0.05).

### Immunohistochemistry

Mouse inner ear sections were harvested, processed and immunostained with anti-Zeb1 or anti-CD326 antibodies as described in Hertzano et al. [13]. CD326 is also known as epithelial cell adhesion/activating molecule (EpCAM) that serves as a specific antigenic marker for epithelial cells [13].

## Supporting Information

Figure S1 Inner ear morphology and hearing thresholds of *Zeb1^ΔEx1^* mice. Paint-filled inner ears of *Zeb1^+/+^*, *Zeb1^ΔEx1/+^*, *Zeb1^ΔEx1/ΔEx1^* and *Zeb1^ΔEx1/Tw^* mice at E14.5 are shown from lateral (A-D), medial (E-H), ventral (I-L) and dorsal (M-P) views. Inner ears from *Zeb1^+/+^* and *Zeb1^ΔEx1/+^* mice appeared similar and normal. *Zeb1^ΔEx1/ΔEx1^* lateral semicircular canals had a subtle constriction (indicated by *) of the midportion of the canal that differed from those observed in *Tw/+* mice (Figure 2). The lateral semicircular canals of *Zeb1^ΔEx1/Tw^* mice did not contain this abnormality and resemble those of *Tw/+* mice. *Zeb1^ΔEx1/+^* mice have normal ABR thresholds (Q). a, anterior semicircular canal; c, cochlear duct; es, endolymphatic sac; l, lateral semicircular canal; p, posterior semicircular canal; s, saccule; u, utricle.
(TIF)

Figure S2 Sequences of novel exons of *Zeb1*, *Zeb1os* and *Zfp438*. Annotated exons are black and novel unannotated exons identified by 5′-RACE, 3′-RACE and RT-PCR analyses are gray. Sequences of novel unannotated exon sequences are shown. For *Zeb1*, alternative first exons 1a, 1b, 1c, 1d, 1e and 1f are each spliced to exon 2.
(TIF)

Figure S3 Southern blot confirmation of homologous recombination and *lacZ*-PGK-Neo^R^ cassette removal of *KI^A^*. A. Genomic DNA was digested with BglII and hybridized with the 3′ probe shown in Figure 5. The probe hybridizes to 4.2- and 5.9-kb fragments before and after Cre-mediated excision of the *lacZ*-PGK-Neo^R^ cassette, respectively. B. Nucleotide sequence confirmation of *KI^G^* and *KI^A^* at c.58+181G/A.
(TIF)

Table S1 PCR primers and probes for mapping, genotyping and real-time PCR. Sequences are shown for PCR primers: 40 novel short tandem repeats located between D18Mit109 and D18Mit201 on mouse chromosome 18; used to genotype knockin mice before (*KI^G:Neo^* and *KI^A:Neo^*) and after (*KI^G^* and *KI^A^*) Cre-mediated excision of the *lacZ*-PGK-Neo^R^ cassette; quantitative RT-PCR analysis of *Zeb1*, *Zeb1os*, *Zfp438* and *Zeb2*. *Actb* and *Gapdh* were included as controls to calculate relative expression.
(TIF)
