# Supplementary figures and images for: A Noncoding Point Mutation of Zeb1 Causes Multiple Developmental Malformations and Obesity in Twirler Mice
Source: PLoS Genet. 2011 Sep 29;7(9):e1002307. doi: 10.1371/journal.pgen.1002307 (PMC3183090; doi:10.1371/journal.pgen.1002307)

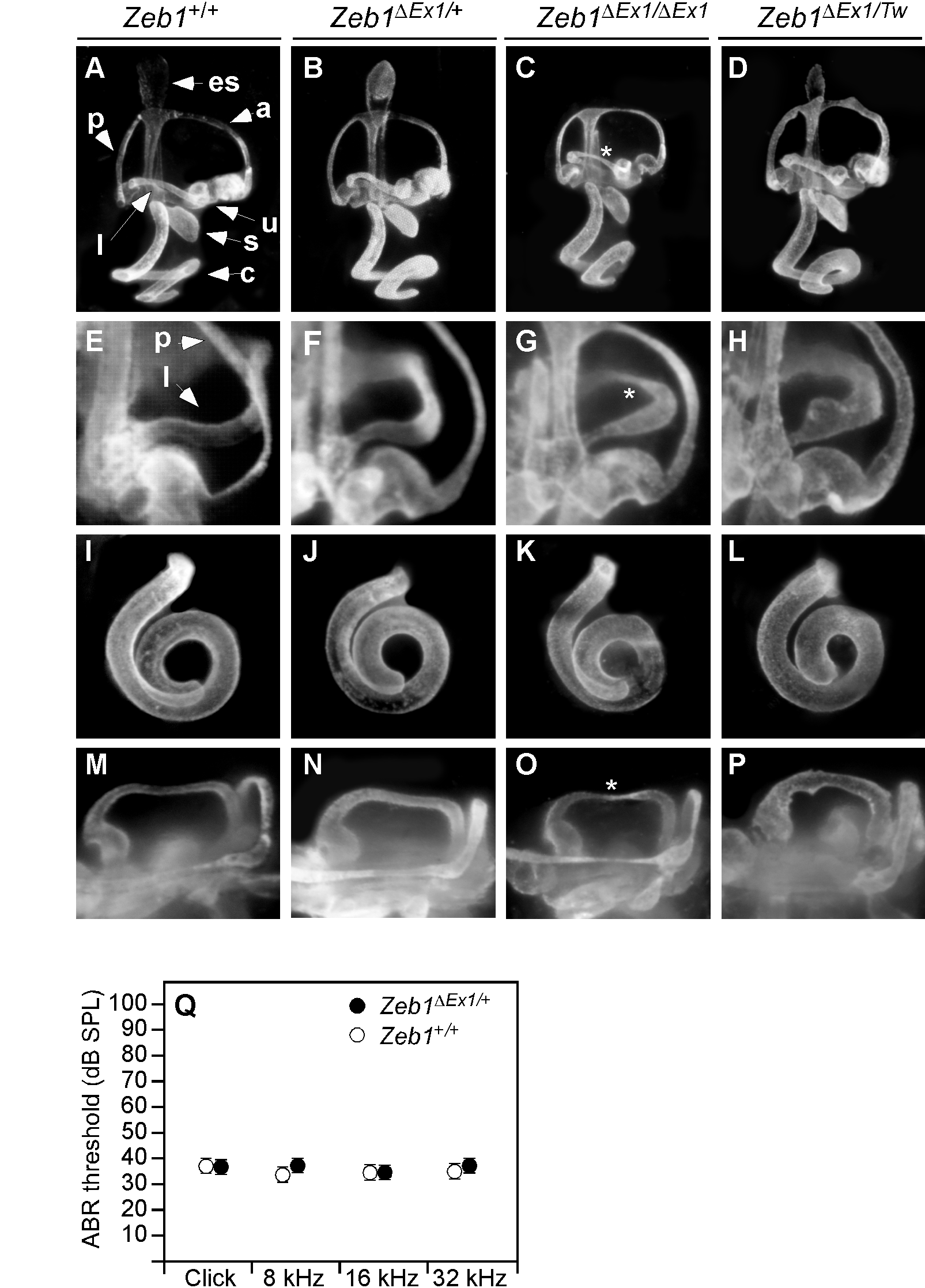

Supplement: Figure S1 — Inner ear morphology and hearing thresholds of Zeb1ΔEx1 mice. Paint-filled inner ears of Zeb1+/+, Zeb1ΔEx1/+, Zeb1ΔEx1/ΔEx1 and Zeb1ΔEx1/Tw mice at E14.5 are shown from lateral (A-D), medial (E-H), ventral (I-L) and dorsal (M-P) views. Inner ears from Zeb1+/+ and Zeb1ΔEx1/+ mice appeared similar and normal. Zeb1ΔEx1/ΔEx1 lateral semicircular canals had a subtle constriction (indicated by *) of the midportion of the canal that differed from those observed in Tw/+ mice (Figure 2). The lateral semicircular canals of Zeb1ΔEx1/Tw mice did not contain this abnormality and resemble those of Tw/+ mice. Zeb1ΔEx1/+ mice have normal ABR thresholds (Q). a, anterior semicircular canal; c, cochlear duct; es, endolymphatic sac; l, lateral semicircular canal; p, posterior semicircular canal; s, saccule; u, utricle. (TIF) [file pgen.1002307.s001.tif]

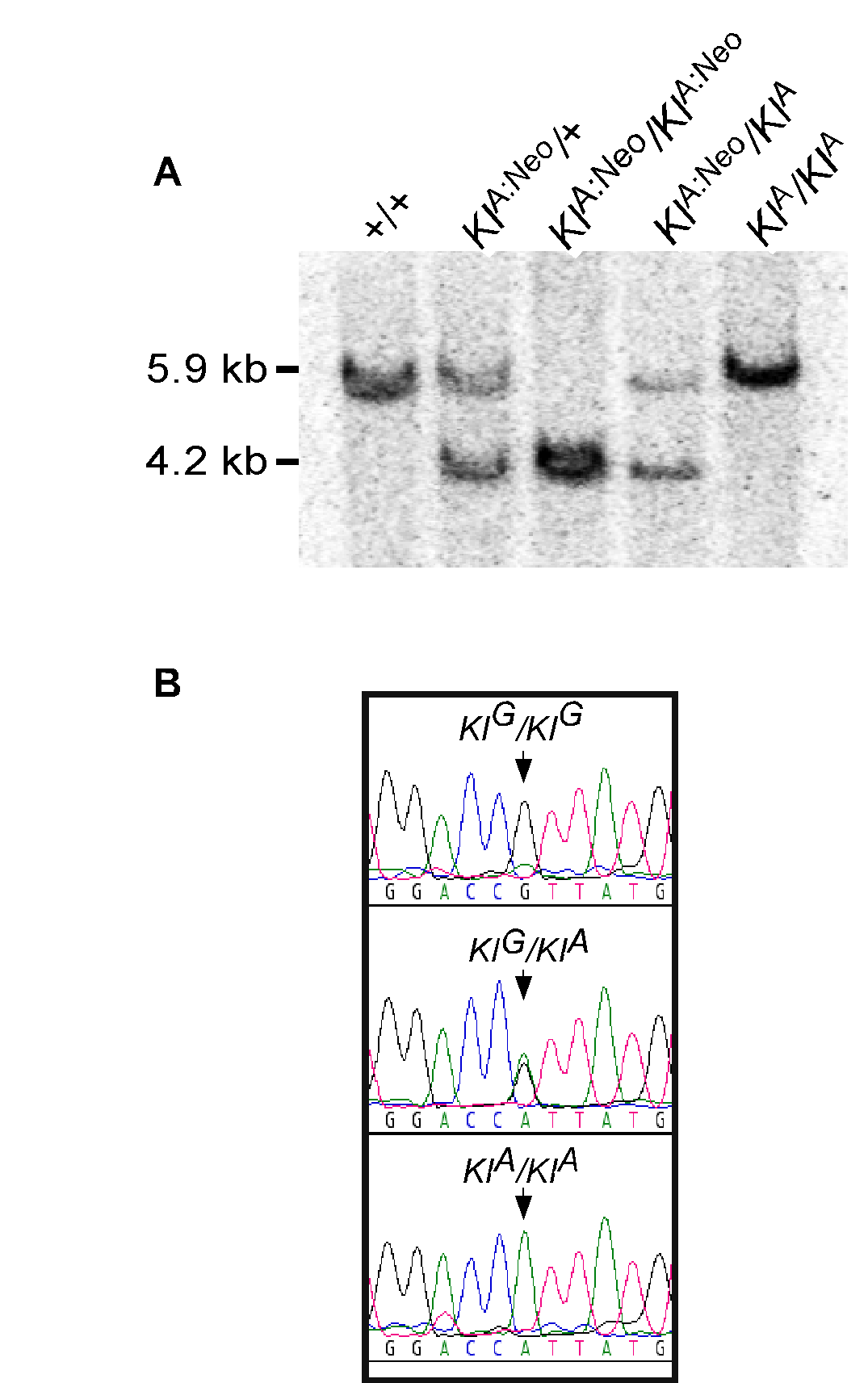

Supplement: Figure S3 — Southern blot confirmation of homologous recombination and lacZ-PGK-NeoR cassette removal of KIA. A. Genomic DNA was digested with BglII and hybridized with the 3′ probe shown in Figure 5. The probe hybridizes to 4.2- and 5.9-kb fragments before and after Cre-mediated excision of the lacZ-PGK-NeoR cassette, respectively. B. Nucleotide sequence confirmation of KIG and KIA at c.58+181G/A. (TIF) [file pgen.1002307.s003.tif]
